# 8Spheres conformal microspheres as embolic agents for symptomatic uterine leiomyoma therapy in uterine artery embolization (UAE): A prospective clinical trial

**DOI:** 10.1097/MD.0000000000033099

**Published:** 2023-03-03

**Authors:** Yiwen Zhang, Yanneng Xu, Xun Zhang, Bo Zheng, Wei Hu, Gang Yuan, Guangyan Si

**Affiliations:** a Department of Interventional Radiology, The Affiliated Traditional Chinese Medicine Hospital of Southwest Medical University, Luzhou, China.

**Keywords:** microsphere, ovarian reserve, symptomatic uterine leiomyoma, uterine artery embolization

## Abstract

To evaluate the treatment efficacy of uterine artery embolization (UAE) using 8Spheres conformal microspheres for symptomatic uterine leiomyoma. In this prospective observational study, 15 patients were enrolled and underwent UAE by 2 experienced interventionalists from September 1, 2018, to September 1, 2019. All patients underwent menstrual bleeding scores, the symptom severity domain of the Uterine Fibroid Symptom and Quality of Life questionnaire scores (with lower scores indicating mild symptoms), pelvic contrast-enhanced magnetic resonance imaging, ovarian reserve tests (estradiol, prolactin, testosterone, follicle-stimulating, luteinizing, and progesterone), and other appropriate preoperative examinations within 1 week before UAE. During follow-up, menstrual bleeding scores and the symptom severity domain of the Uterine Fibroid Symptom and Quality of Life questionnaire scores were recorded at 1, 3, 6, and 12 months after UAE to assess the efficacy of symptomatic uterine leiomyoma. Pelvic contrast-enhanced magnetic resonance imaging was performed 6 months after the interventional therapy. Biomarkers of ovarian reserve function were reviewed at 6 and 12 months after treatment. All 15 patients successfully underwent UAE, without severe adverse effects. Six patients experienced abdominal pain, nausea, or vomiting, all of which improved significantly after symptomatic treatment. The menstrual bleeding scores declined from baseline (350.2 ± 61.9 mL) to (131.8 ± 42.7 mL), (140.3 ± 42.4 mL), (68.0 ± 22.8 mL), and (64.43 ± 17.0 mL) at 1, 3, 6, and 12 months, respectively. The symptom severity domain scores at 1, 3, 6, and 12 months postoperatively were significantly lower and statistically significant compared to the preoperative scores. The uterus and dominant leiomyoma volumes decreased from baseline (340.0 ± 35.8 cm^3^), (100.6 ± 24.3 cm^3^) to (266.6 ± 30.9 cm^3^), (56.1 ± 17.3 cm^3^) at 6 months after UAE, respectively. Moreover, the ratio of leiomyoma volumes and uterus decreased from (27.4 ± 4.5%) to (18.7 ± 3.9%). At the same time, there was no significant effect on changes in the biomarkers of ovarian reserve levels. Only the changes in testosterone levels before and after UAE were statistically significant (*P* < .05). 8Spheres conformal microspheres are ideal embolic agents for UAE therapy. This study showed that 8Spheres conformal microsphere embolization for symptomatic uterine leiomyoma could effectively relieve heavy menstrual bleeding, improve the symptom severity of patients, reduce the volume of leiomyoma, and have no significant effect on ovarian reserve function.

## 1. Introduction

Uterine leiomyomas are the most common gynecological tumors,^[1]^ and their lifetime prevalence exceeds approximately 70% among women.^[2]^ Uterine leiomyomas can cause heavy or prolonged menstrual bleeding and anemia in women of reproductive age, seriously affecting their quality of life.^[3]^ Large leiomyomas and enlarged uteri can also result in pressure symptoms (e.g., as bowel and bladder dysfunction, abdominal protrusion, and infertility.^[1]^

Hysterectomy is the traditional approach for the management of symptomatic leiomyomas.^[4]^ However, adverse events are common in patients who undergo hysterectomy, with a 28% risk of medical or surgical complications (e.g., significant blood loss, wound complications, and febrile episodes) and a 10% risk of transfusion.^[1]^ Moreover, a study showed substantial subsequent medical complications after hysterectomy, including the risks of heart disease and mental health conditions, even conserving both ovaries.^[5]^ Myomectomy,^[4]^ high-intensity focused ultrasound (HIFU) ablation,^[6]^ and uterine artery embolization (UAE)^[7]^ are standard methods for uterine-sparing interventions during the treatment of leiomyomas. In general, myomectomy is best suited for treating one–three dominant leiomyoma inaccessible locations. However, one-fifth of patients who undergo myomectomy require a second surgery at 5 years.^[8]^ Currently, patients with more than 4 leiomyomas are not considered good candidates for HIFU therapy, and a retrospective study showed the best results in leiomyomas less than 50 mL in volume.^[9]^ UAE is increasingly used as a minimally invasive treatment for uterine leiomyomas.^[10]^ Compared with myomectomy, UAE has the advantages of relatively less trauma and shorter hospital stay.^[11]^ Compared to HIFU, UAE has the advantage of a lower reintervention rate.^[12]^

8Spheres conformal microspheres (Suzhou Hengrui Callisyn Biomedical Technology Co., Ltd, China) were used to embolize benign tumors.^[13]^ As is well known, novel microspheres are not used as embolic agents in UAE. Herein, we designed a prospective single-arm study to further evaluate the efficacy of 8Spheres conformal microspheres in the UAE. In this study, the primary outcomes were changes in menstrual bleeding scores and leiomyoma volume before and after UAE. The secondary outcomes included changes in the symptom severity domain of the Uterine Fibroid Symptom and Quality of Life (UFS-QOL) questionnaire scores and ovarian reserve (estradiol, prolactin, follicle-stimulating, luteinizing, and progesterone hormones) before and after treatment.

## 2. Materials and Methods

### 2.1. Study design

This prospective clinical trial was approved by the institutional review board. Written informed consent for the procedure and opt-out consent for the retrospective use of data were obtained from all patients. No funding related to the present study was received from organizations or sponsors.

### 2.2. Inclusion and exclusion criteria for UAE

Enrolled patients met the following inclusion criteria: patients will, premenopausal, age ≥ 20 years, symptomatic intramural uterine leiomyoma (e.g., including menorrhagia, pain, or bulk-related symptoms), menstrual volume score ≥ 150 mL, diameter of leiomyoma less than 10 cm, and no further fertility requirements. The exclusion criteria included gynecologic malignancies, an undiagnosed pelvic mass, acute pelvic infection, coagulopathy, pregnancy, menopause, contrast allergy, and completely infarcted dominant leiomyoma.

### 2.3. Preoperative preparation

Patients were sufficiently rested, ensured sufficient sleep time, and maintained emotional stability. They were eating high-protein foods to supplement their daily protein and calorie requirements. Liver and kidney function, coagulation function, ovarian reserve biomarkers, and other routine tests were performed before interventional therapy. The Allen test was performed to assess whether a transradial artery puncture could be achieved.

### 2.4. UAE treatment protocol

8Spheres conformal microspheres as embolic agents were used to embolize the leiomyoma in all enrolled patients. The procedures were usually performed 3 to 7 days after the menstrual period. The technique of “free flow embolization” was employed to perform all procedures by a 5-Fr catheter (Radifocus Introducer II; Terumo Corporation, Tokyo, Japan) combined with a coaxially introduced microcatheter (Stride 2.6-Fr microcatheter; ASAHI INTEC CO, Tokyo, Japan). Radiologists with 10 years of interventional therapy experience performed the UAE. All patients underwent UAE *via* right radial artery access. First, a 5-Fr single curved catheter bypassed the arch of the aorta, passed through the descending aorta and abdominal aorta, and was finally selected into the internal iliac artery. The uterine artery was then superselected using a microcatheter. After angiography to determine the leiomyoma supply arteries, microspheres with sizes of 500 to 700 μm or 700 to 900 μm were slowly injected into the target arteries under fluoroscopic guidance. The endpoint was defined as complete occlusion of the supply arteries, with contrast remaining in the blood vessels for 6 to 8 cardiac cycles. Next, the procedure was repeated for the contralateral uterine artery.

### 2.5. Postoperative treatment

The puncture site required compression 6 hours after UAE, and patients were administered anti-infective and symptomatic treatments. Postoperative follow-up was carried out by telephone or outpatient visits for 12 months to observe the occurrence of postoperative syndrome.

### 2.6. MRI examinations

All patients underwent pelvic contrast-enhanced magnetic resonance imaging (MRI) (MAGNETOM Skyra 3.0 T; SIEMENS Healthineers) within 1 week preoperatively and 6 months postoperatively. The rats were fasted for 4 hours before the examination. The bladder was filled with moderate urine, and the patient was placed in the supine position. Fast spin-echo T2-weighted and T1-weighted gradient-echo imaging was performed. Contrast-enhanced MRI was performed 45 seconds after the intravenous infusion of 10 mL of gadolinium chelate. The MRI system with its post-processing workstation, with 2 experienced radiologists to review the images and observe the number of leiomyomas, location, and signal change of T1-weighted gradient-echo during contrast enhancement. The volume of the dominant uterine leiomyomas and uterus was calculated using the formula (ABC × 0.5236 cm^3^, where A, B, and C represent the length, width, and height).

### 2.7. Data collection

All patients underwent menstrual bleeding scoring, symptom severity domain of the UFS-QOL questionnaire (with lower scores indicating mild symptoms), pelvic contrast-enhanced MRI, ovarian reserve tests, and other appropriate preoperative examinations within 1 week before UAE. During follow-up, menstrual bleeding scores and the symptom severity domain of the UFS-QOL questionnaire scores were recorded at 1, 3, 6, and 12 months after UAE. Pelvic contrast-enhanced MRI was performed 6 months after the interventional therapy. Ovarian reserve function biomarkers were reviewed at 6 and 12 months after treatment.

### 2.8. Statistical analysis

Data are presented as the mean ± standard error of the mean (SEM), based on at least 3 independent experiments. Tukey’s post hoc test was used to evaluate statistical differences using 1-way analysis of variance. Statistical significance was set at *P* < .05. software (GraphPad Prism version 8.0.1; GraphPad, San Diego, CA).

## 3. Results

### 3.1. Participant characteristics

Fifteen patients with uterine leiomyomas were admitted to the Affiliated Traditional Chinese Medicine Hospital of Southwest Medical University between September 1, 2018, and September 1, 2019. All 15 patients in this trial completed the treatment successfully, and 6 patients had abdominal pain, nausea, or vomiting, all of which improved significantly after symptomatic treatment, and no patient had severe adverse effects. The baseline characteristics of all patients are shown in Table 1.

**Table 1:** Baseline characteristics.

Characteristic	Value
Number of patients	15
Age, yr	41.5 ± 6.7
Weight, kg	59.4 ± 7.2
Body mass index	24.2 ± 2.8
Symptoms	
Menorrhagia	15 (100%)
Pain	12 (80.0%)
Bulk-related symptoms	9 (60.0%)
UFS-QOL total score	100.1 ± 9.7
Uterine volume, mL	340.0 ± 138.6
Dominant tumor volume, mL	100.6 ± 94.2
Location of dominant tumor	
Intramural	15 (100%)
Technical success	15 (100%)

UAE = uterine artery embolization.

### 3.2. Menstrual bleeding scores

**Figure 1.**
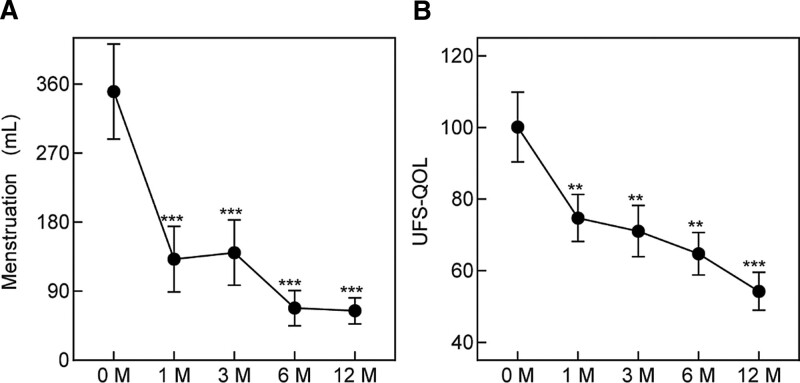
Menstrual bleeding scores and symptom severity scores before and after UAE. (A) The menstrual bleeding scores were performed before UAE. After UAE, the scores were performed again at 1, 3, 6, and 12 mo. (B) The symptom severity of the UFS-QOL questionnaire scores assessed before UAE. After UAE, the scores were reevaluated at 1, 3, 6, and 12 mo. The data shown represent the mean ± SEM n = 15, **P < .01, ***P < .001. UAE = uterine artery embolization, UFS-QOL = Uterine Fibroid Symptom and Quality of Life.

All 15 patients had heavy menstrual bleeding (350.2 ± 61.9 mL) at baseline. Menstrual bleeding scores were collected again at 1, 3, 6, and 12 months after UAE to assess the efficacy of symptomatic uterine leiomyoma. The menstrual bleeding scores were decline to (131.8 ± 42.7 mL), (140.3 ± 42.4 mL), (68.0 ± 22.8 mL), and (64.43 ± 17.0 mL) at 1, 3, 6, and 12 months, respectively (Fig. 1A). There were statistically significant differences (*P* < .001) in menstrual bleeding scores before and after UAE, which tended to be stable after 6 months.

### 3.3. Symptom severity scores

The score on the symptom severity domain of the UFS-QOL questionnaire was used to assess improvement in uterine leiomyoma symptoms. Before UAE, the symptom severity scores were (100.1 ± 9.7), while the scores decreased to (74.7 ± 6.5), (71.0 ± 7.2), (64.7 ± 5.9), and (54.3 ± 5.3) at 1, 3, 6, and 12 months, respectively (Fig. 1B). There were statistically significant differences (*P* < .01) in symptom severity scores before and after UAE.

### 3.4. Changes in uterus and dominant leiomyoma volumes

**Figure 2.**
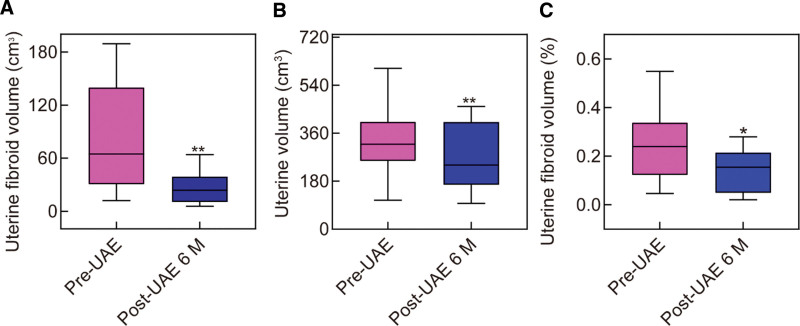
Changes in dominant leiomyoma (A), uterus volumes (B), and their ratio (C). The data shown represent the mean ± SEM n = 15, *P < .05, **P < .01. UAE = uterine artery embolization.

After UAE at 6 months, MR was rescanned in 15 patients. The uterus and dominant leiomyoma volumes were decreased from (340.0 ± 35.8 cm^3^), (100.6 ± 24.3 cm^3^) to (266.6 ± 30.9 cm^3^), (56.1 ± 17.3 cm^3^), respectively (Fig. 2A and B). At the same time, their ratio shrunk from (27.4 ± 4.5%) to (18.7 ± 3.9%) (Fig. 2C). Analysis of volume changes after UAE showed a significant reduction in uterine volume, dominant leiomyoma volume, and their ratio (*P* < .05).

### 3.5. Biomarkers of ovarian reserve

**Figure 3.**
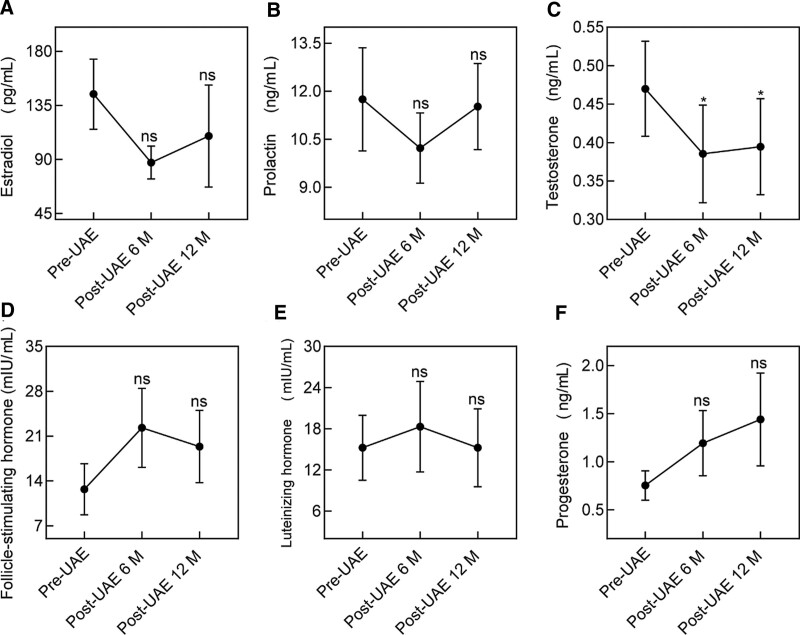
The changes of ovarian reserve biomarkers (estradiol, prolactin, testosterone, follicle-stimulating, luteinizing, and progesterone) at baseline, 6 and 12 mo have been shown in this figure. The data shown represent the mean ± SEM n = 15, *P < .05, ns > 0.05. UAE = uterine artery embolization.

Only the changes in testosterone levels were statistically significant (*P* < .05) before and after UAE, whereas there was no significant effect on the changes in other biomarkers of ovarian reserve (Fig. 3), indicating no significant impact on ovarian reserve after UAE for uterine leiomyomas. We divided all patients into 2 groups based on age to compare the postoperative changes in ovarian reserve function. The older group is older or equal to 40 years, while others were classified as the younger group. There was no statistically significant difference in any biomarker between the 2 groups at baseline (*P* > .05). At 6 and 12 months after UAE, only the testosterone level of the older group was significantly lower than that of the younger group (*P* < .05). These results showed that changes in testosterone levels after UAE were correlated with age. Therefore, it can be inferred that UAE has no effect on pregnancy among women of appropriate age.

## 4. Discussion

UAE is a well-established treatment for uterine leiomyomas.^[7]^ A critical step in UAE is the choice of the best embolic agent. Non-spherical or spherical polyvinyl alcohol (PVA) particles (Bearing; Merit Medical, and Contour SE; Boston Scientific) and Tris-acryl gelatin microspheres (TAGM) (Embosphere Microspheres; Merit Medical) has used for embolization of uterine leiomyomas during interventional therapy.^[14,15]^ Compared to spherical PVA and TAGM, particulate PVA does not pass through a microcatheter during injection.^[7]^ Particulate PVA trendily adheres to the vessel wall, causing obstruction of the proximal vessels and affecting their dispersion in the malformed vascular mass, which easily induces the establishment of collateral circulation and leads to recanalization of the malformed vessels.^[15]^ In addition, particulate PVA results in a more significant inflammatory response.^[14]^ Spherical PVA was assumed to be equivalent to TAGM when it was introduced into practice. However, subsequent studies have shown surprisingly poor outcomes with low rates of leiomyoma infarction.^[16]^ TAGM has been widely used as the first spherical material in the UAE. However, in a high-quality study, there were no differences in the rates of complete dominant leiomyoma necrosis, use of rescue analgesics, symptom severity score, and health-related quality-of-life score between the TAGM and particulate PVA groups after embolization.^[14]^ Thus, novel embolic materials are strongly desired for clinical practice in UAE. 8Spheres conformal microspheres (Suzhou Hengrui Callisyn Biomedical Technology Co., Ltd, China) were used to embolize benign tumors.^[13]^ It is well known that all 8Spheres conformal microspheres are not used in UAE as embolic agents.

In our study, the menstrual bleeding score was one of the primary outcomes. At the same time, heavy menstrual bleeding is also the main clinical symptom of uterine leiomyomas. Heavy menstrual bleeding was defined as >80 mL of blood loss per menstrual cycle for at least 2 separate cycles.^[17]^ Previous studies have suggested that UAE can effectively relieve the symptoms of menorrhagia, and the corresponding guidelines recommend UAE for the treatment of uterine leiomyomas causing menorrhagia.^[18]^ In this group of patients, the improvement in menstrual blood loss at 6 months after UAE gradually stabilized. Therefore, the results indicate that the 8Spheres conformal microspheres can achieve the ideal effect in UAE.

Furthermore, the postoperative leiomyoma volume and uterine volume were reduced to different degrees in all 15 patients compared with the preoperative volume, and the differences were statistically significant (*P* < .01). Moreover, in the MRI examination 6 months after UAE, the enhancement of uterine leiomyomas was diminished in all patients compared to the preoperative period. Meanwhile, the symptom severity domain of the UFS-QOL questionnaire showed significant improvement in patients after UAE. Thus, this trial also confirmed the effectiveness and safety of 8Spheres conformal microspheres for uterine leiomyoma embolization.

Ovarian reserve function may significantly negatively impact future quality of life and morbidity, such as an increased risk of osteoporosis, cardiovascular disease, and all-cause mortality.^[19]^ According to a previous study, ovarian reserve appears to be affected by UAE in premenopausal women, whereas younger ovaries exhibit a greater capacity for recovery after ovarian damage.^[20]^ In addition, a systematic review of 15 randomized controlled trials and prospective cohort studies showed that loss of ovarian function after embolization occurred mainly in women over 45 years of age.^[21]^ When patients are older, residual ovarian function is limited, and tolerance to possible damaging factors (e.g., reduced blood flow, misembolization) is low. Thus, subclinical effects on ovarian function manifest in patients nearing menopause, leading to amenorrhea. To further evaluate the effect of UAE on ovarian reserve function, we compared changes in biomarkers preoperative and postoperative. Except for testosterone, we did not find any effect of UAE on the ovarian reserve function. In addition, we found that with an age cutoff of 40 years, the older group had lower testosterone levels than the younger group, and the difference was statistically significant, suggesting that age may be a relevant factor in UAE affecting ovarian function. However, the absence of statistically significant changes in testosterone levels in the younger group of patients also does not mean that ovarian reserve function is completely unaffected in any way. The study of ovarian reserve function relies on the analysis of serum hormone levels, including FSH, E2, anti-Müllerian hormone, and testosterone. Recent studies indicated that women with diminished ovarian function and poor responders benefit from concurrent androgen supplementation.^[22,23]^ Nevertheless, it is controversial whether these protocols are necessary. In the study by Xiao et al^[24]^ who found that pregnancy outcomes were not affected by testosterone levels, they also found that as testosterone levels decreased with age, lower serum testosterone levels were correlated with decreases in the numbers of retrieved oocytes and available embryos, indicating that testosterone levels were closely related to ovarian aging.

There were also some limitations to this study and some deviations in the interpretation of the results.

1. The number of cases included in this study was small and lacked randomized controlled validation.
2. The follow-up period of this study was only 1 year, which is a short follow-up period. There is no follow-up on whether the patients have an asymptomatic recurrence in the long term, and whether there are other reinterventions after myoma enlargement.
3. The 15 patients included in this study did not desire fertility after UAE. Therefore, there are no objective follow-up results on whether UAE affects fertility.

It is hoped that further studies with increased sample sizes and follow-up times will be conducted in greater depth.

## 5. Conclusion

In conclusion, 8Spheres conformal microspheres are ideal embolic agents for UAE. This study showed that 8Spheres microsphere embolization for symptomatic uterine leiomyoma can effectively relieve heavy menstrual bleeding, improve the symptom severity of patients, reduce the volume of leiomyoma, and have no significant effect on ovarian reserve function.

## Acknowledgments

The authors are thankful to the Department of Interventional Radiology, The Affiliated Traditional Chinese Medicine Hospital of Southwest Medical University, for their support in this study.

## Author contributions

**Conceptualization:** Yanneng Xu, Guangyan Si.

**Data curation:** Yiwen Zhang, Yanneng Xu, Xun Zhang, Bo Zheng, Wei Hu.

**Formal analysis:** Yiwen Zhang, Bo Zheng.

**Investigation:** Xun Zhang, Wei Hu, Gang Yuan.

**Methodology:** Xun Zhang, Gang Yuan.

**Software:** Yanneng Xu.

**Supervision:** Bo Zheng.

**Writing – original draft:** Yiwen Zhang.

**Writing – review & editing:** Yanneng Xu, Guangyan Si.
